# Construction of a prognostic signature for serous ovarian cancer based on lactate metabolism-related genes

**DOI:** 10.3389/fonc.2022.967342

**Published:** 2022-09-15

**Authors:** Jiangdong Xiang, Rongjia Su, Sufang Wu, Lina Zhou

**Affiliations:** ^1^ Department of Obstetrics and Gynecology, Shanghai General Hospital, Shanghai Jiaotong University School of Medicine, Shanghai, China; ^2^ Department of Gynecologic Oncology, International Peace Maternity and Child Health Hospital, Shanghai Jiaotong University School of Medicine, Shanghai, China

**Keywords:** ovarian cancer, lactate metabolism‐related genes, prognostic signature, overall survival, the cancer genome atlas

## Abstract

**Background:**

The key biochemical feature of malignant tumor is the conversion of energy metabolism from oxidative phosphorylation to glycolysis, which provides sufficient capacity and raw materials for tumor cell rapid growth. Our study aims to construct a prognostic signature for ovarian cancer based on lactate metabolism-related genes (LMRGs).

**Methods:**

Data of ovarian cancer and non-diseased ovarian data were downloaded from TCGA and the GTEx database, respectively. LMRGs were obtained from GeneCards and MSigDB databases, and the differentially expressed LMRGs were identified using limma and DESeq2 R packages. Cox regression analysis and LASSO were performed to determine the LMRGs associated with OS and develop the prognostic signature. Then, clinical significance of the prognostic signature in ovarian cancer was assessed.

**Results:**

A total of 485 differentially expressed LMRGs in ovarian tissue were selected for subsequent analysis, of which 324 were up-regulated and 161 were down regulated. We found that 22 LMRGs were most significantly associated with OS by using the univariate regression analysis. The prognostic scoring model was consisted of 12 LMRGs (SLCO1B3, ERBB4, SLC28A1, PDSS1, BDH1, AIFM1, TSFM, PPARGC1A, HGF, FGFR1, ABCC8, TH). Kaplan-Meier survival analysis indicated that poorer overall survival (OS) in the high-risk group patients (P<0.0001). This prognostic signature could be an independent prognostic indicator after adjusting to other clinical factors. The calibration curves of nomogram for the signature at 1, 2, and 3 years and the ROC curve demonstrated good agreement between the predicted and observed survival rates of ovarian cancer patients. Furthermore, the high-risk group patients have much lower expression level of immune checkpoint-TDO2 compared with the low-risk group (P=0.024).

**Conclusions:**

We established a prognostic signature based on LMRGs for ovarian cancer, and highlighted emerging evidence indicating that this prognostic signature is a promising approach for predicting ovarian cancer prognosis and guiding clinical therapy.

## Introduction

Ovarian cancer is one of the most lethal reproductive system malignant tumors that seriously threatens women’s health in the world. Epithelial ovarian cancer accounts for about 85% - 90%, and its mortality is the highest among gynecological malignant tumors (1). The characteristics of early invasion and metastasis of ovarian cancer are the main reasons for the poor prognosis and high mortality. Most patients were already in advanced stage when they were first diagnosed with ovarian cancer and lost the best opportunity for treatment. Recently, although some progress has been made in chemotherapy and biotherapy of ovarian cancer, the five-year survival rate of patients is still low, hovering below 50% (2). Therefore, there is an urgent need to develop more reliable and more effective biomarkers for the early detection, diagnosis, prognosis prediction and monitoring of ovarian cancer.

Compared with normal cells, a key biochemical feature of malignant tumor cells is the conversion of energy metabolism from oxidative phosphorylation to aerobic glycolysis (3, 4). Metabolic reprogramming is recognized as a hallmark of ovarian cancer, whereby cancer cells exhibit high glycolytic flux with excessive lactate production, even under adequate oxygen conditions (5, 6). A large amount of lactate produced by glycolysis in the tumor microenvironment (TME) was previously considered to be only a metabolic waste. However, in recent years, more and more studies have proved that lactate can play a role in promoting tumor process (7, 8). The production and accumulation of lactate cause the pH 6-6.6 acidic tumor environment which is associated with increased tumor metastasis, angiogenesis, recurrence, treatment resistance and poor prognosis (9–11). Accordingly, targeting aerobic glycolysis, especially lactate, seems become a promising therapeutic approach for cancer.

In ovarian cancer, suppression of glucose consumption and lactate production, and therefore inhibition of the Warburg effect, exert the anti-tumor effect (12, 13). Lactate also controlled VEGF-A/VEGFR2 expression and the resulting cell invasion in ovarian cancer (14). Other researchers suggested that lactate is a reliable predictor of ICU length of stay following ultra-radical ovarian cancer surgery. Early recognition and correction of hyperlactatemia following advanced ovarian cancer may reduce ICU length of stay, limiting both the resource pressure and patient morbidity/mortality sequelae (15). Similarly, our previous studies found that the increased expression of LDH, a key enzyme regulating lactate metabolism, was associated with high metastasis, high invasion and low survival rate of ovarian cancer (16, 17). It is generally believed that lactate plays an important role in the pathogenesis and progression of ovarian cancer, targeting its metabolism is expected to become an effective means of cancer treatment. However, few studies have comprehensively analyzed the relationship between lactate metabolism-related genes (LMRGs) and the prognosis, and survival of ovarian cancer.

In the present study, we screened the key lactate metabolism-related genes (LMRGs) and constructed a prognostic signature to explore an efficient metabolic biomarker for the more accurate stratification management of ovarian cancer. A nomogram that combined 12 LMRGs was created for predicting the 1-, 3- and 5-year OS of ovarian cancer, and Cox regression analysis was applied to identify the prognostic value and clinical relationship of the signature in ovarian cancer.

## Materials and methods

### Data collection

The RNA-seq data, including 427 patients with ovarian cancer downloaded from The Cancer Genome Atlas (TCGA; http://cancergenome.nih.gov/) database and 88 non-diseased ovarian tissue downloaded from the GTEx (Genotype-Tissue Expression) database were analyzed. Search and download 4725 lactate metabolism-related genes from GeneCards database and 32 lactate metabolism-related genes from MSigDB (http://www.gsea-msigdb.org/gsea/msigdb/search.jsp) database.

### Differentially expressed LMRGs in ovarian cancer

The differentially expressed lactate metabolism-related genes (LMRGs) in ovarian cancer and normal ovarian tissues were screened and analyzed using limma and DESeq2 R packages (18, 19). |logFC|>1 and adjusted P<0.05 were considered as significant and were selected for subsequent analysis. Using PCA (principal component analysis) to identify the differential expression genes (DEGs) and investigate the differences between ovarian cancer and normal ovarian tissue. Then, using the ggplot 2, pheatmap and ggbiplot packages in R to plot volcano map, heat map and PCA map for the DEGs.

### Construction of the lactate metabolism-related prognostic scoring model for ovarian cancer

The 411 ovarian cancer samples with survival information in the TCGA dataset were obtained as the training set for constructing the prognosis risk model, and the 153 ovarian cancer samples with survival information in the GSE26712 dataset were explored for external validation. LMRGs associated with the OS of patients with ovarian cancer were identified by using univariate Cox analysis, and only LMRGs with a P value < 0.05 were selected for subsequent analysis. LASSO Cox regression was carried out to avoid the prognostic model overfitting and narrow the genes for prediction of the OS. LMRGs detected *via* LASSO algorithm were evaluated by multivariate Cox regression analysis. The LMRGs prognostic scoring model was established based on the expression level of each gene and its corresponding regression coefficient, shown as risk score = ∑ (βi × Expi), where βi represented the corresponding regression coefficient of a gene and Expi represented the expression level of a gene (20). Patients were divided into low-risk group and high-risk group based on the risk score formula with the median risk score as the cut-off point. The Kaplan-Meier (K-M) and log-rank test were used to calculate the differences in overall survival (OS) between the two groups. The receiver operating characteristic (ROC) curve was implemented and the corresponding area under the ROC curve (AUC) was measured to assess the sensitivity and specificity of the prognostic scoring model.

### Association of the prognostic scoring model and clinicopathological features

We used univariate and multivariate Cox regression analyses to estimate the effect of the risk score on OS and the clinicopathologic features (age, clinical stage, histological grade, and lymphatic metastasis). The correlation between the expression of these LMRGs and several clinical features were also explored. In addition, the accuracy of the prediction between the risk score and the clinicopathologic features was compared by time-dependent ROC curve.

### Assessing the immuno‐/chemotherapeutic response of the risk subtypes for ovarian cancer patients

Immune checkpoints play an important role in tumor immune escape, and immune checkpoint therapy has made important clinical progresses and provided a new weapon against cancer (21, 22). Therefore, the proportion of immune cells infiltrate in TME in the sample was calculated and the expression of crucial immunomodulators were investigated between low- and high-risk groups. Download GSE102073 clinical data from GEO dataset, which contains 233 samples including platinum-based chemotherapy sensitivity. The survival curve of platinum resistant and sensitive groups was drawn by ggsurvplot package in R. The risk score of each ovarian cancer patients between platinum resistant and sensitive groups was shown by ggboxplot package in R.

### Statistical analysis

All statistical analyses and visualization were performed by version 4.1.3 of R software (https://www.r-project.org) and SPSS (version 19.0; SPSS Company, Chicago, IL). The Mann-Whitney U test was used to compare the two groups. The Kaplan-Meier survival curves were compared with the log-rank test. The immune scores were calculated by ESTIMATE package. LASSO Cox proportional hazards regression analysis was performed by penalized package. For all tests, data were considered to be statistically significant with two-sided P value < 0.05.

## Results

### Identification of differentially expressed LMRGs in ovarian cancer

**Figure 1:**
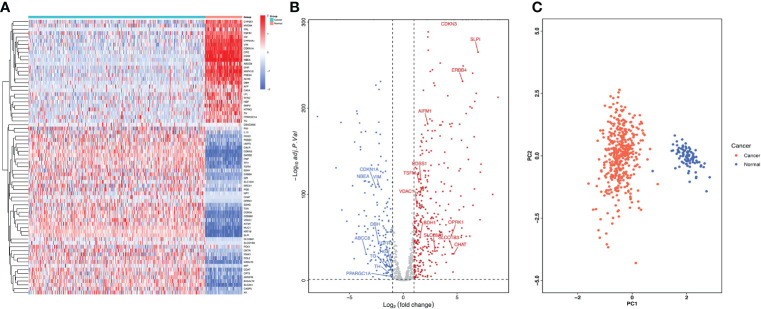
Differentially expressed LMRGs in ovarian cancer in the TCGA dataset. **(A)** Heatmap of LMRGs between ovarian cancer and normal ovarian tissues, the color from blue to red represents the progression from low expression to high expression. **(B)** Volcano plot of LMRGs, the red dots in the plot represents upregulated genes and blue dots represents downregulated genes with statistical significance. Gray dots represent no differentially expressed genes. **(C)** PCA map of DEGs related to lactate metabolism distinguishing ovarian cancer from normal tissue.

We examined the differentially expressed genes (DEGs) related to lactate metabolism using the R package DESeq2, considering the cut-off criteria (adjusted P<0.05 and |log FC|>1.0), 485 differentially expressed LMRGs in ovarian tissue were selected for subsequent analysis, of which 324 were up-regulated and 161 were down regulated (**Figures 1A, B**). The PCA map indicated that these LMRGs can well distinguish ovarian cancer from normal ovarian tissue (**Figure 1C**).

### Identification of LMRGs associated with prognosis of patients with ovarian cancer

**Figure 2:**
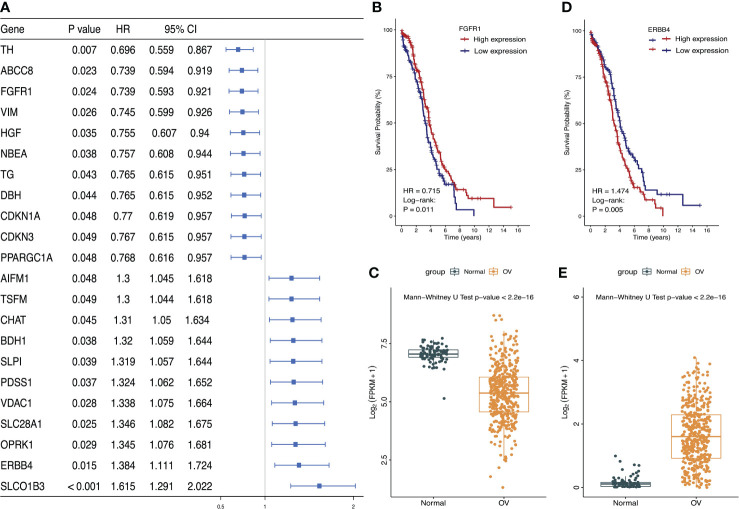
Identified the most significant LMRGs related to ovarian cancer risk. **(A)** Identification of LMRGs associated with ovarian cancer patient prognosis based on univariate Cox regression analysis. **(B, C)** Survival analysis of FGFR1 and its expression level in ovarian cancer and normal ovarian tissues. **(D, E)** Survival analysis of ERBB4 and its expression level in ovarian cancer and normal ovarian tissues. P < 0.05 was considered to be statistically significant.

Univariate Cox proportional hazard regression analysis was performed on 485 differentially expressed LMRGs. The result showed that 22 LMRGs were significantly associated with patient prognosis (**Figure 2A**, P<0.05). The forest map was drawn for these LMRGs, of which 11 LMRGs HR < 1 and 11 LMRGs HR > 1. Select a gene with HR < 1 to draw the K-M survival curve, and showed the survival rate of FGFR1 high-expression group was much higher than FGFR1 low-expression group (**Figure 2B**, P=0.011), moreover, the expression level of FGFR1 in normal ovarian tissues was notably higher than that in ovarian cancer tissues (**Figure 2C**; P<0.001). Then, select a gene with HR > 1 to draw the K-M survival curve, and the result showed that the survival rate of ERBB4 high-expression group was significantly lower than ERBB4 low-expression group (**Figure 2D**, P=0.005), OS was inversely correlated with ERBB4 expression levels. Furthermore, the expression level of ERBB4 in normal ovarian tissues was significantly lower than that in ovarian cancer tissues (**Figure 2E**, P<0.001).

### Establishment and validation of the lactate metabolism‐related prognostic signature for ovarian cancer

**Figure 3:**
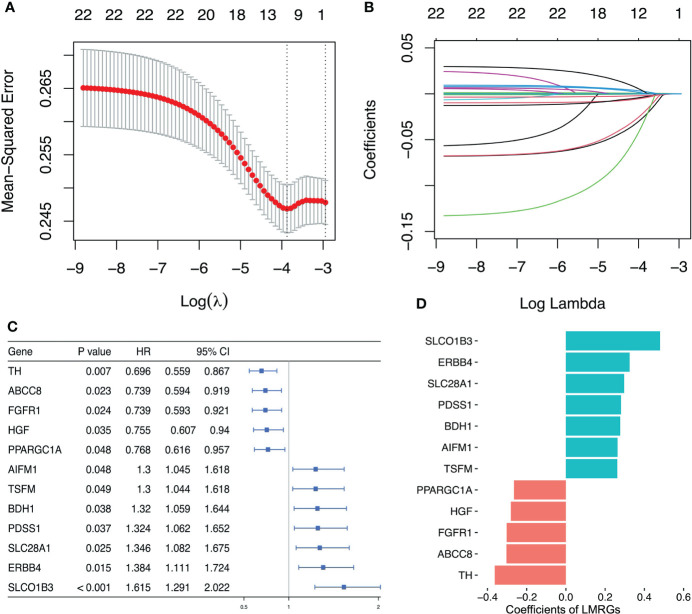
Establishment of lactate metabolism-related prognostic risk signature. **(A)** Screening of optimal λ value in LASSO model at which the vertical lines were drawn. **(B)** LASSO coefficient profiles of the 22 LMRGs with non-zero coefficients determined by the optimal λ value. **(C)** Multivariate cox analysis to developing a prognostic signature based on these LMRGs. **(D)** Coefficients of the 12 LMRGs.

Based on the results of univariate Cox regression analysis, 22 lactate metabolism related genes with the most significant difference were selected to construct LASSO regression mode, and then using cv.glmnet function to obtain the optimal λ (lambda) (**Figure 3A**). Lasso coefficient profiles of the 22 LMRGs with non-zero coefficients determined by the optimal lambda (**Figure 3B**). Finally, a prognostic signature was established based on multivariate Cox regression analysis, and 12 LMRGs (ERBB4, FGFR1, SLC28A1, BDH1, PPARGC1A, ABCC8, SLCO1B3, PDSS1, AIFM1, TSFM, HGF, TH) were confirmed to establish the prognostic scoring model, of which 5 LMRGs HR < 1 and 7 LMRGs HR > 1 (**Figure 3C**). The coefficients of PPARGC1A, HGF, FGFR1, ABCC8 and TH were < 0, and the coefficients of SLCO1B3, ERBB4, SLC28A1, PDSS1, BDH1, AIFM1 and TSFM were > 0 (**Figure 3D**). We calculated the LMRGs signature score of every ovarian cancer patient based on the coefficient and the expression of the 12 crucial LMRGs, shown as follows: Risk score = (0.480×expression value of SLCO1B3) + (0.325×expression value of ERBB4) + 0.297×expression value of SLC28A1) + (0.281×expression value of PDSS1) + (0.277×expression value of BDH1) + (0.263× expression value of AIFM1) + (0.262×expression value of TSFM) + (- 0.265×expression value of PPARGC1A) + (- 0.281×expression value of HGF) + (-0.302×expression value of FGFR1) + (- 0.303×expression value of ABCC8) + (- 0.363×expression value of TH).

**Figure 4:**
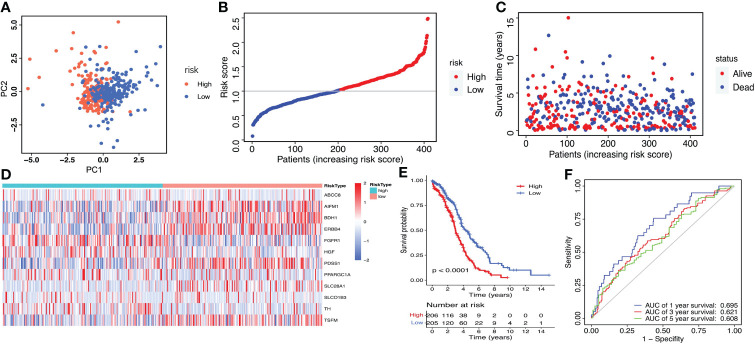
Construction of the LMRGs-based prognostic risk signature in the TCGA dataset. **(A)** PCA was used to determine whether the samples could be grouped correctly based on the prognostic risk signature. **(B)** The risk score distribution of patients with ovarian cancer. **(C)** Survival status of patients with ovarian cancer. **(D)** Heatmap of the 12 LMRGs expression. **(E)** Survival curves for the low- and high-risk groups. **(F)** ROC curves analysis of the risk signature on OS at 1 year, 3 years, and 5 years.

Then, the risk score of each patient was calculated according to this prognostic model. Taking the median risk score as cut-off, a total of 411 ovarian cancer patients were classified into a high-risk group (n=206) and a low-risk group (n=205) (**Figure 4A**). The risk score and survival status of each ovarian cancer patient were presented in **Figures 4B, C**. The heat map showed the differential expression of the 12 crucial LMRGs in the two risk subgroups (**Figure 4D**). Kaplan-Meier survival analysis indicated that patients in the high-risk group showed markedly poorer OS than those in the low-risk group (P<0.0001, **Figure 4E**). AUC values of the risk score model predicting the 1-, 3- and 5-year OS rates were 0.695, 0.621 and 0.608 respectively, indicating that this prognostic model exhibited a good sensitivity and specificity and has potential value in predicting the prognosis of patients with ovarian cancer (**Figure 4F**).

**Figure 5:**
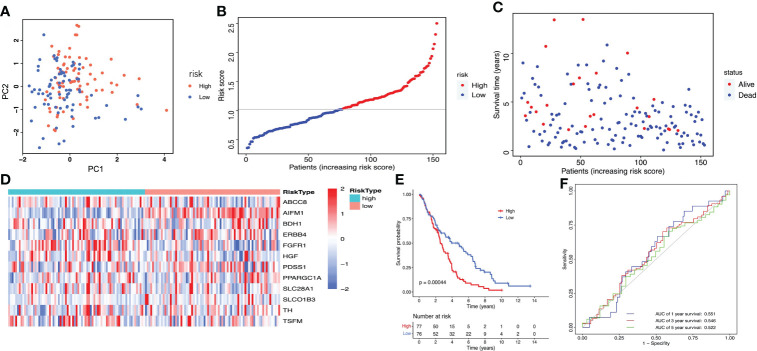
Validation of the LMRGs-based prognostic risk signature in the GSE26712 dataset. **(A)** PCA was used to determine whether the samples could be grouped correctly based on the prognostic risk signature. **(B)** The risk score distribution of patients with ovarian cancer. **(C)** Survival status of patients with ovarian cancer. **(D)** Heatmap of the 12 LMRGs expression. **(E)** Survival curves for the low- and high-risk groups. **(F)** ROC curves analysis of the risk signature on OS at 1 year, 3 years, and 5 years.

The GSE26712 dataset including 153 ovarian cancer samples were used for the external validation of the lactate metabolism-related signature. According to the median risk score, we divided patients into high-risk (n=77) and low-risk groups (n=76) (**Figure 5A**). The risk score and survival status of every ovarian cancer patient were displayed in **Figures 5B, C**. The heat map showed the differential expression of the 12 crucial LMRGs in the high- and low-risk subgroups (**Figure 5D**). Consistent with the results derived from the TCGA database, the Kaplan-Meier curve demonstrated that patients in the high-risk group exhibited markedly poorer OS than those in the low-risk group (**Figure 5E**), and the AUC values of the risk score model predicting the 1-, 3- and 5-year OS rates were 0.551, 0.546 and 0.522 respectively, also indicating that this prognostic model has potential value in predicting the prognosis of patients with ovarian cancer (**Figure 5F**).

### The prognostic signature is an independent prognostic factor for ovarian cancer

**Figure 6:**
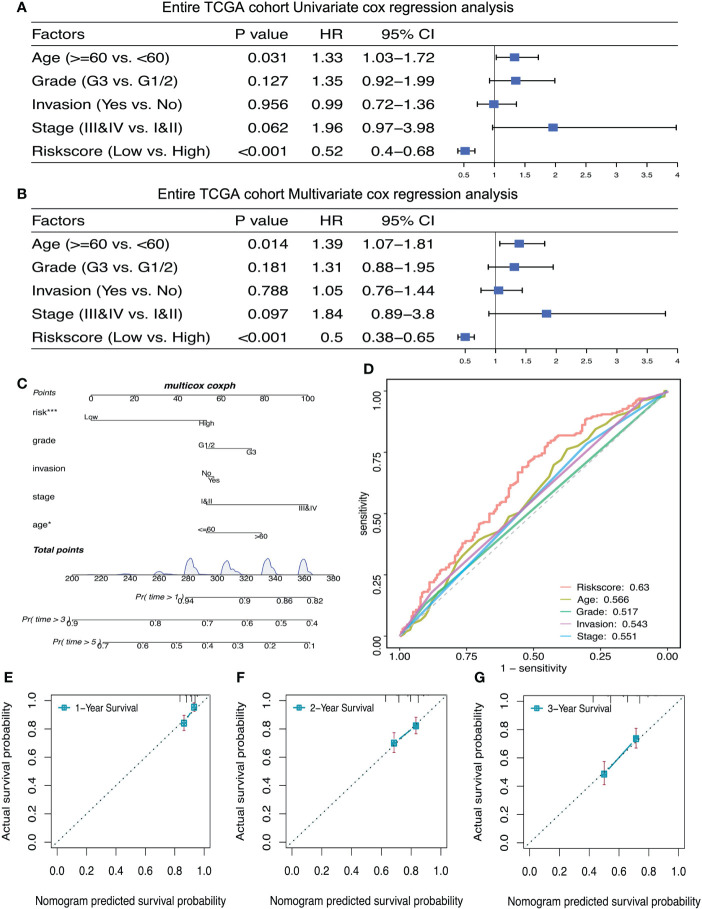
The prognostic risk signature was an independent prognostic factor of ovarian cancer. **(A, B)** Univariate and multivariate Cox regression analyses of independent risk factors for OS in patients with ovarian cancer. **(C)** Nomogram for independent risk factors for OS in patients with ovarian cancer. D: AUC to verify the independent risk factors. **(E–G)** Calibration curves of nomogram for the prognostic scoring model at 1, 2, and 3 years. *P < 0.05, ***P < 0.001.

Univariate and Multivariate Cox regression analysis were used to verify whether lactate metabolism‐related prognostic signature is an independent prognostic factor. After adjusting other clinical features, including age, cancer stage, lymphatic invasion and histochemical score, the risk scoring model can be used as an independent prognostic factor of ovarian cancer (**Figures 6A, B**). Bootstrap method for repeated sampling is used for internal validation of the prognostic signature, and the result indicated that the model has a good degree of discrimination (**Figure 6C**). The ROC curve and the calibration curves of nomogram for the signature at 1, 2, and 3 years demonstrated that the predicted results of nomogram are in good agreement with the actual survival rates for each of OS (**Figures 6D–G**).

### Assessment of the immuno‐/chemotherapeutic response in the risk subtypes for ovarian cancer patients

**Figure 7:**
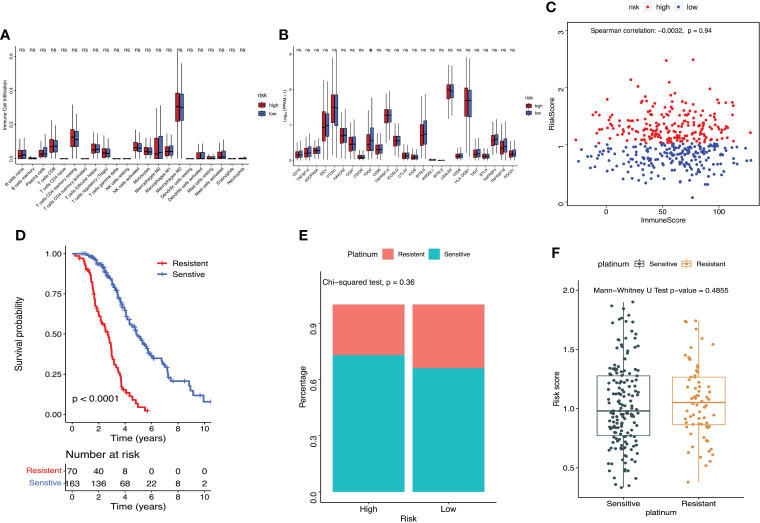
Assessment of the immuno‐/chemotherapeutic response in the risk subtypes for ovarian cancer patients in the TCGA dataset. **(A)** Proportion of immune cell infiltration in TME between high- and low-risk groups. **(B)** Expression of immune checkpoints between high- and low-risk groups. **(C)** ESTIMATE calculated the correlation between the prognostic risk signature and immune score. **(D)** K-M survival curve of platinum-sensitive and platinum-resistant patients. **(E)** Percentages of platinum sensitivity in high- and low-risk groups. **(F)** Risk scores of platinum-sensitive and platinum-resistant patients. *P < 0.05. ns, no significance.

Cibersort was used to calculate the proportion of immune cells in TME. Boxplot showed that there was no significant difference in the proportion of immune cell infiltration between groups with high- and low-risk scores (**Figure 7A**). The immune checkpoint TDO2 gene showed much higher expression level in the low-risk group compared with the high-risk group (P=0.024, **Figure 7B**). Using ESTIMATE package in R to calculate immune score of the sample, and Spearman correlation assessment to evaluate the correlation between lactate metabolism‐related risk score and immune score, and the result showed no correlation (**Figure 7C**). The survival rate of ovarian cancer patient in platinum sensitive group was dramatically higher (P<0.0001, **Figure 7D**). Compared with the low-risk group (66%), the platinum sensitive ovarian cancer patients in the high-risk group were considerably increased (73%), although there was no significant difference between the two groups (P=0.36, **Figure 7E**). There was no significant difference in risk scores between platinum sensitive and resistant groups (P=0.48, **Figure 7F**).

## Discussion

One of the key biochemical characteristics of malignant tumors is metabolic reprogramming, which promotes glucose uptake and enables tumor cells to choose glycolysis as the main energy source even under the condition of normal oxygen content. A large amount of lactate production in this process provides the needs for rapid growth. Lactate, produced by glycolysis, keeps the acidic microenvironment of tumor cells locally, which is conducive to the invasion, metastasis and drug resistance of tumor cells, and is related to the poor prognosis (7–10, 23). In fact, lactate plays an important role in the pathogenesis and progression of ovarian cancer, thus, targeting its metabolism is expected to become a promising therapeutic approach.

In this study, 485 DEGs related to lactate metabolism were identified through differential expression analysis, 324 of which significantly up-regulated while 161 of which significantly down-regulated in ovarian cancer. Based on the results of Cox regression analysis, 22 LMRGs with the most significant differences were selected to logistic LASSO regression. Through LASSO analysis, twelve DEGs (ERBB4, FGFR1, SLC28A1, BDH1, PPARGC1A, ABCC8, SLCO1B3, PDSS1, AIFM1, TSFM, HGF, TH) related to lactate metabolism were used to construct a risk score model and used as a prognostic signature for ovarian cancer. ERBB (Erb-B2 Receptor Tyrosine Kinase) family members are often overexpressed, amplified, or mutated in many forms of cancer, making them important therapeutic targets (24). Among them, the role of ERBB4 in cancer remains controversial (25). It was found that high-level ERBB4 expression was observed at a significantly higher frequency in ovarian serous carcinoma compared with normal control tissue, indicated that ERBB4 expression may correlate with chemotherapy-resistant ovarian serous carcinoma and shortened OS (26). Inhibitors targeting ERBB4 may be broadly applicable as therapeutic agents in majority of cancers (27). FGFR1 (Fibroblast Growth Factor Receptor 1) is overexpressed in the majority of human tumors, including ovarian cancer (28). FGFR1 activation contributes to aerobic glycolysis and transformation of epithelial cells or reprograming the energy metabolism of cancer cells. FGFR1 inhibition impacts on cancer cell growth by affecting glucose energy metabolism, and could be an important therapeutic option across multiple tumor types (29–31). Study found that a high expression of SLC28A1 (Solute Carrier Family 28 Member 1) was significantly associated with poor overall survival in pancreatic cancer patients (32). BDH1(3-Hydroxybutyrate Dehydrogenase 1) is a key enzyme that regulates the metabolism and synthesis of ketone bodies. Studies found that expression of BDH1 was also associated with decreased time to biochemical relapse and decreased progression-free survival (33). PPARGC1A (PPARG Coactivator 1 Alpha), also known as PGC-1α, is highly expressed in ovarian cancer cells, some researchers showed its high expression in cisplatin-resistant cells (34), while some others revealed its overexpression could induce cell apoptosis (35). These findings provide strong theoretical support for PGC1α as a potential therapeutic target in ovarian cancer. Study showed that ABCC8 (ATP Binding Cassette Subfamily C Member 8) is an independent prognostic factor for glioma, which can predict chemosensitivity, and patients with high expression of ABCC8 have longer survival time (36).

Based on the LMRGs prognostic scoring model, ovarian cancer patients were assigned into low-risk group and high-risk group with the median risk score as the cut-off point. It was found that the prognostic signature has good potential value in predicting the prognosis of patients with ovarian cancer. K-M curve showed significant higher survival probability in low-risk group compared with high-risk group, high risk scores meant poor prognosis. Heatmap revealed that the expression levels of the 12 LMRGs in ovarian cancer tissues of high- and low-risk groups were significantly different. Univariate and multivariate Cox regression analysis verified that the risk model can be used as an independent prognostic factor of ovarian cancer. The calibration curves of nomogram for the signature at 1, 2, and 3 years and the ROC curve demonstrated that the predicted results of nomogram are in good agreement with the actual survival rates of ovarian cancer patients for each of OS, which can be used to guide clinical treatment. Similarly, other researchers also constructed prognostic signatures for hepatocellular and renal carcinoma based on LMRGs (20, 37). Nevertheless, no one use LMRGs to build signature for ovarian cancer by now. Someone used glycolysis-related gene or energy metabolism−related gene or microenvironment-related genes to construct signature for survival prediction of ovarian cancer patients (38–40). Compared with our study, although these studies use different types and different quantity of genes, all the signatures can well predict the prognosis of ovarian cancer.

Chemotherapy is the classic treatment for ovarian cancer, and immunotherapy is the hotspot in ovarian cancer research. In the current study, the correlation between lactate risk score and immune infiltration and platinum sensitivity was also preliminarily explored. Studies highlighted emerging evidence that lactate in the TME can exert immunosuppressive function, and promote the development of tumor by inducing and recruiting a plethora of immunosuppressive related cells and molecules (41–43). Our study suggested that there was no difference in the proportion of immune cell infiltration in TME between the high- and low-risk groups, but the immune checkpoint TDO2 was closely correlated with the risk score model, and its expression was significantly higher in the high-risk group. It is reported that TDO2 is highly expressed in many tumors and promotes tumor progression, and as a promising cancer treatment target, it has attracted more and more attention. Reports revealed that inhibition of TDO2 can repress the proliferation, migration and invasion of ovarian cancer and colorectal cancer (44, 45). The prognostic signature was probably associated with TDO2 expression and TDO2 checkpoint pathway in ovarian cancer. In addition, we found that the survival rate of ovarian cancer patients was dramatically higher in the platinum sensitive group. There were more platinum-sensitive ovarian cancer patients in the high-risk group, and the risk score of platinum-resistant patients was much higher, although there was no significant difference between the two groups. Besides, there was no significant difference in risk scores between the platinum sensitive and resistant groups. Although our results do not provide strong evidence, the prognostic scoring model may become a promising strategy for evaluating the response of immunotherapy and platinum-based chemotherapy in ovarian cancer.

In this study, twelve LMRGs as prognostic indicators were identified for the first time to be possibly associated with the survival outcome of ovarian cancer. We found that the lactate metabolism-related prognostic scoring model, as an independent prognostic factor, has good potential in predicting the prognosis of patients with ovarian cancer. To our knowledge, this is the first time to construct a signature with LMRGs to predict the prognosis of ovarian cancer. However, there are still some limitations in the present study. First, no *in vitro* or *in vivo* molecular experiment was performed to verify our analysis. Second, the robustness of the prognostic scoring model must be verified in large prospective studies in the future. Third, our study was a retrospective study, so, prospective study is in need to validate the findings of our study in the future.

## Conclusion

Collectively, in this study we identified a novel prognostic scoring model based on LMRGs that could be applied to predict the prognosis of patients with ovarian cancer. This prognostic signature is an independent prognostic indicator of ovarian cancer, and may provide valuable information either for diagnosis or developing novel therapeutic options for ovarian cancer patients in the future.

## Data availability statement

The original contributions presented in the study are included in the article/supplementary material. Further inquiries can be directed to the corresponding author.

## Author contributions

JX and LZ are responsible for experimental design. RS is responsible for instrument operation. RS and SW are responsible for data analysis. JX and LZ are for providing overall ideas. All authors contributed to the article and approved the submitted version.

## Funding

The present study was supported by the Clinical characteristic medical technology cultivation plan of Shanghai General Hospital (no. 02.DY12.06.22.07 to LZ and no. 02.DY12.06.22.12 to JX).

## Conflict of interest

The authors declare that the research was conducted in the absence of any commercial or financial relationships that could be construed as a potential conflict of interest.

## Publisher’s note

All claims expressed in this article are solely those of the authors and do not necessarily represent those of their affiliated organizations, or those of the publisher, the editors and the reviewers. Any product that may be evaluated in this article, or claim that may be made by its manufacturer, is not guaranteed or endorsed by the publisher.
